# Effects of Mo Addition on Microstructure and Corrosion Resistance of Cr_25-x_Co_25_Ni_25_Fe_25_Mo_x_ High-Entropy Alloys via Directed Energy Deposition

**DOI:** 10.3390/mi15101196

**Published:** 2024-09-27

**Authors:** Han-Eol Kim, Jae-Hyun Kim, Ho-In Jeong, Young-Tae Cho, Osama Salem, Dong-Won Jung, Choon-Man Lee

**Affiliations:** 1 Department of Smart Manufacturing Engineering, Changwon National University, 20 Changwondaehak-ro, Uichang-gu, Changwon-si 51140, Republic of Korea; 20237177@gs.cwnu.ac.kr (H.-E.K.); ytcho@changwon.ac.kr (Y.-T.C.); 2 Mechatronics Research Center, Changwon National University, Changwon 51140, Republic of Korea; jaehyunkim@changwon.ac.kr (J.-H.K.); jhi8443@changwon.ac.kr (H.-I.J.); 3 Department of Production Engineering and Mechanical Design, Faculty of Engineering, Menoufia University, Shebin El-Kom 6132711, Egypt; osama.mohammed@sh-eng.menofia.edu.eg; 4 Faculty of Applied Energy System, Major of Mechanical Engineering, Jeju National University, 102 Jejudaehak-ro, Jeju-si 63243, Republic of Korea

**Keywords:** directed energy deposition, additive manufacturing, material property, high entropy alloy, corrosion resistance

## Abstract

Highly entropy alloys (HEAs) are novel materials that have great potential for application in aerospace and marine engineering due to their superior mechanical properties and benefits over conventional materials. NiCrCoFe, also referred to as Ni-based HEA, has exceptional low-temperature strength and microstructural stability. However, HEAs have limited corrosion resistance in some environments, such as a 3.5 wt% sodium chloride (NaCl) solution. Adding corrosion-resistant elements such as molybdenum (Mo) to HEAs is expected to increase their corrosion resistance in a variety of corrosive environments. Metal additive manufacturing reduces production times compared to casting and eliminates shrinkage issues, making it ideal for producing homogeneous HEA. This study used directed energy deposition (DED) to create Cr_25-x_Co_25_Ni_25_Fe_25_Mo_x_ (x = 0, 5, 10%) HEAs. Tensile strength and potentiodynamic polarization tests were used to assess the materials’ mechanical properties and corrosion resistance. The mechanical tests revealed that adding 5% Mo increased yield strength (YS) by 20.1% and ultimate tensile strength (UTS) by 9.5% when compared to 0% Mo. Adding 10% Mo led to a 32.5% increase in YS and a 20.4% increase in UTS. Potentiodynamic polarization tests were used to assess corrosion resistance in a 3.5-weight percent NaCl solution. The results showed that adding Mo significantly increased initial corrosion resistance. The alloy with 5% Mo had a higher corrosion potential (E_corr_) and a lower current density (I_corr_) than the alloy with 0% Mo, indicating improved initial corrosion resistance. The alloy containing 10% Mo had the highest corrosion potential and the lowest current density, indicating the slowest corrosion rate and the best initial corrosion resistance. Finally, Cr_25-x_Co_25_Ni_25_Fe_25_Mo_x_ (x = 0, 5, 10%) HEAs produced by DED exhibited excellent mechanical properties and corrosion resistance, which can be attributed to the presence of Mo.

## 1. Introduction

High-entropy alloys (HEAs) have gained popularity in aerospace and marine applications as a result of their superior mechanical properties, which surpass those of conventional materials [1,2]. Conventional alloys have been beaten by HEAs, as demonstrated by extensive research conducted over the past decade. These include the capacity to maintain their structure and properties at elevated temperatures, a robust resistance to microstructural changes, high hardness, and exceptional high-temperature strength. Furthermore, HEAs demonstrate outstanding resistance to high-temperature softening, corrosion, fatigue, and wear [3].

In contrast to conventional alloys, HEAs are composed of five or more elements, each of which contributes 5–35% to the overall composition [4]. The high mixing entropy of these alloys is the consequence of the uniform mixing of various elements. This high entropy results in the formation of a variety of crystal structures, such as single-phase face-centered cubic (FCC) or body-centered cubic (BCC) structures, as well as mixed structures that contain multiple phases. It is possible to design a variety of HEAs with exceptional properties at extremely low temperatures, such as superior corrosion resistance and strengths that surpass those of conventional alloys [5]. 

CrMnFeCoNi, a type of Cantor alloy, is known for its exceptional strength in cryogenic environments, rendering it an optimal choice for low-temperature applications [6]. HEAs with high chromium (Cr) and molybdenum (Mo) content are gaining popularity in marine engineering due to their exceptional corrosion resistance, which is critical. HEAs exhibit outstanding structural stability and mechanical properties as a result of their slow diffusion rate and lattice strain effect. The lattice strain effect, which is induced by atomic size mismatches when combining various elements, enhances the strength and resistance to deformation [7]. The microstructure remains stable at elevated temperatures due to the slow diffusion rate, which improves both corrosion resistance and low-temperature strength.

Additive manufacturing (AM) is a process that involves the layer-by-layer deposition of wire or metal powder using a high-energy heat source, such as a laser or electron beam [8]. AM techniques can be classified into two main groups: directed energy deposition (DED) and powder bed fusion (PBF). PBF employs a laser to target specific regions of metal powder on a substrate [9]. DED includes the simultaneous introduction of metal powder or wire into the laser module from the side [10]. 

AM overcomes issues related to the blending and distribution of alloying components that can occur during conventional casting techniques for HEAs. Conventional casting often causes an uneven distribution of alloying elements during the solidification process, leading to areas with different compositions and properties. Furthermore, variations in the cooling rates between the interior and exterior during the casting process can lead to flaws such as porosity and irregular grain boundaries. 

AM techniques, such as selective laser melting, allow for careful control of material composition and distribution, assuring a more homogeneous and consistent microstructure. This technique eliminates the flaws commonly linked to conventional casting techniques. Researching the manufacturing of HEAs using AM is essential due to these advantages [11]. 

HEAs are manufactured with AM that provides superior mechanical properties and stability while saving both time and money. Additionally, removing constraints on specific shapes or structures facilitates a more customizable structural design technique. In the aerospace and marine industries, molybdenum (Mo)-added alloys are often used due to their exceptional corrosion resistance. 

Chemical stability and corrosion resistance are enhanced by the incorporation of Mo into CrCoNiFe-based HEAs. In NaCl solutions, Dai et al. created FeCoCrNiMox alloys with varying Mo proportions and investigated their corrosion behavior and mechanisms [12]. The environmental corrosion behavior of HEAs was examined by Fu et al., who established a correlation between corrosion resistance, microstructure, and composition. 

This study improved our understanding of HEA corrosion in a variety of environments and provided critical insights for optimizing HEA performance [13]. Wang et al. used AM to produce CoCrFeNiMo_0.2_ HEA and investigated its microstructure, corrosion properties, and mechanical properties [14]. Gao et al. prepared HEAs by casting Mo into Co_30_Cr_30_(FeNi)_40-x_Mo_x_ (x = 0, 2, 4, 6, 8, 10%) and analyzing the resulting microstructural and mechanical properties [15]. 

Niu et al. cast and annealed CoCrFeNiMo_x_ (x = 0, 0.2, 0.5, 0.8, 1) HEAs to investigate their microstructure, mechanical properties, and corrosion resistance in a NaCl environment [16]. Shang et al. produced homogeneous CoCrFeNiMo_x_ HEAs using the electromagnetic stirring method and investigated their corrosion behavior in NaCl and H_2_SO_2_ solutions to simulate neutral and acidic conditions [17]. 

However, research utilizing powder-based DED with the inclusion of Mo is still relatively few. The utilization of DED instead of other AM techniques belongs to its various advantages. DED enables precise control of material deposition, facilitating the fabrication of complicated shapes and the capacity to repair or add improvements to already present components. Moreover, the ability of DED to handle a wide variety of materials, including high-melting-point metals such as Mo, makes it especially well-suited for applications that demand improved mechanical characteristics and resistance to wear [18].

**Figure 1:**
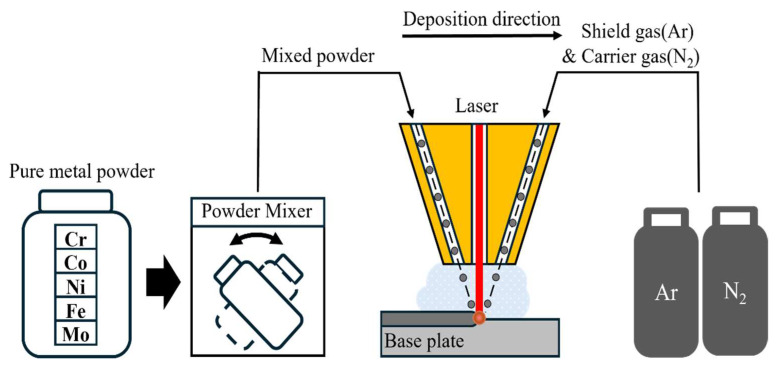
The schematic diagram for the DED process [19].

This study aims to examine the impact of Mo addition on the mechanical properties and corrosion resistance of high-entropy alloys (HEAs) that are manufactured using the DED process. Mo is substituted for manganese (Mn), which is sensitive to saltwater, in the five-element Cantor HEA. Figure 1 illustrates the DED process for CrCoNiFeMo.

**Figure 2:**
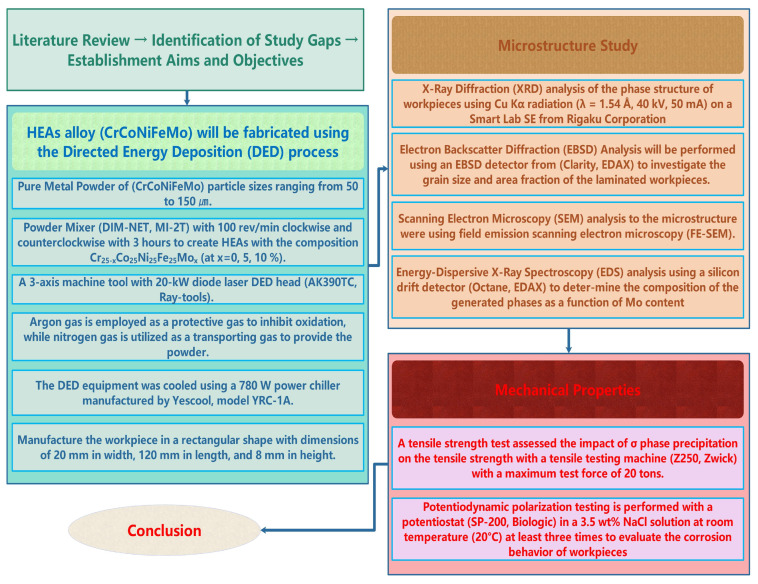
Schematic illustration of the overall research work.

As previously described in a study [16], laminations were conducted with 0%, 5%, and 10% Mo to examine the effect of Mo addition. The laminates’ microstructure, tensile properties, and potentiodynamic polarization behavior were examined using X-ray diffraction (XRD), electron backscatter diffraction (EBSD), scanning electron microscopy (SEM), energy-dispersive X-ray spectroscopy (EDS), and potentiodynamic polarization tests at varying concentrations. The research methodology employed in this investigation is illustrated in Figure 2.

## 2. Directed Energy Deposition (DED) Process

### 2.1. Experimental 

**Figure 3:**
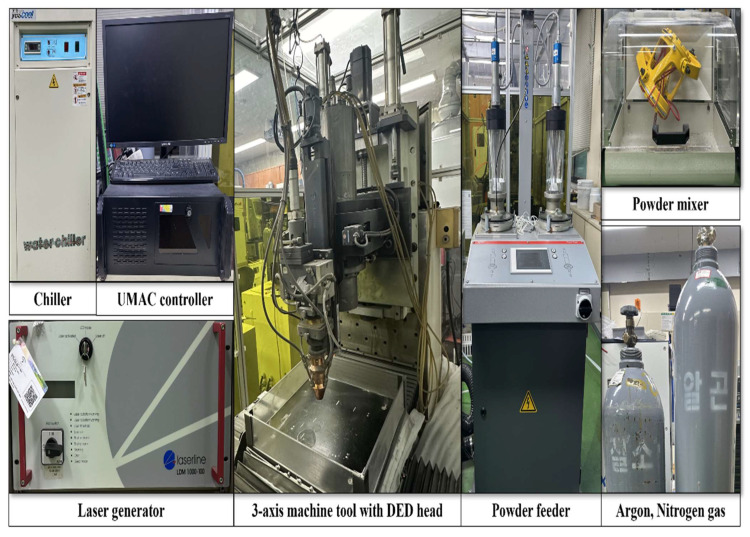
The experimental setup for the DED process.

Figure 3 depicts the experimental setup used in the directed energy deposition (DED) process to create high-entropy alloys (HEAs). A three-axis machine tool with a 20 kW diode laser DED head (AK390TC, Raytools, Burgdorf, Switzerland) was used. The diode laser (LDM1000, Laserline, Mülheim-Kärlich, Germany) has a maximum power output of 1 kW, a focal length of 198 mm, and a 3 mm spot size. The machine tool’s travel range is 500 mm on the *x*-axis, 500 mm on the *y*-axis, and 300 mm on the *z*-axis.

The powder was blended with a powder mixer (DIM-NET, MI-2T, Incheon, Republic of Korea) at 100 revolutions per minute in both clockwise and counterclockwise directions. A 1.5 L capacity twin-disc powder feeder (Twin 150, Oerlikon Metco, Westbury, NY, USA) was used to feed the blended powder into the DED head’s nozzle. The powder was transported and inserted into a nozzle with three holes. Argon gas was used as a protective gas to prevent oxidation, and nitrogen gas was used to transport the powder. The DED equipment was cooled by a 780 W power chiller (Yescool model YRC-1A, Bucheon, Republic of Korea). The experiment’s integrated control was handled by a universal machine and automation controller. To prevent oxidation caused by external air, the DED testing equipment was enclosed.

### 2.2. Materials and Experiment 

**Figure 4:**
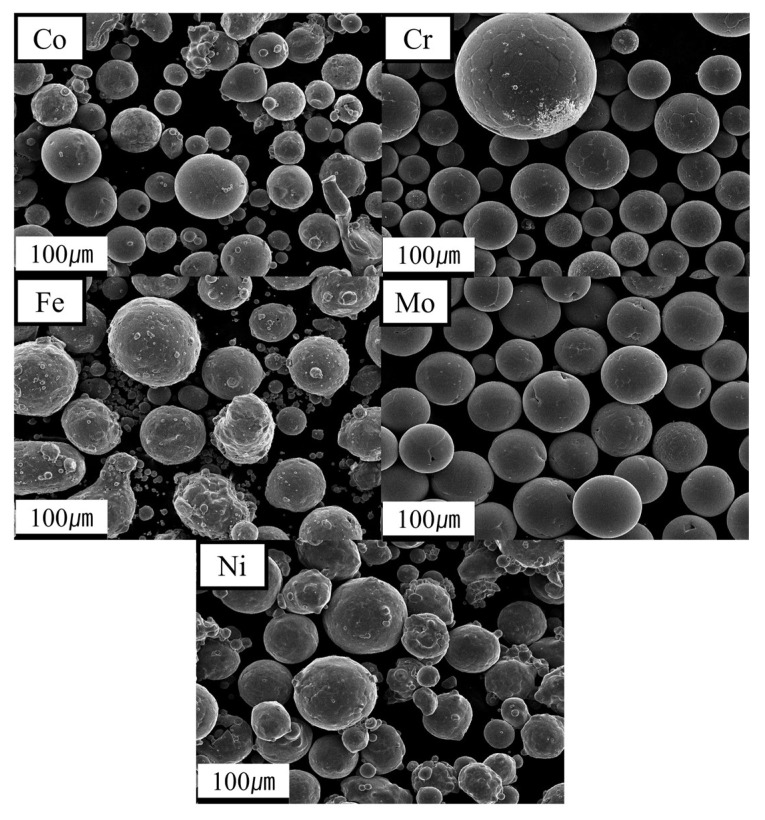
The SEM images of Cr, Co, Ni, Fe, and Mo powder.

The investigation used spherical powders of pure Cr, Co, Ni, Fe, and Mo obtained from Mk Corporation, Vadodara, India, with particle sizes ranging from 50 to 150 µm. The SEM images of these powders are shown in Figure 4. The decision to choose pure metal powder materials instead of CoCrFeNi pre-alloyed powder and pure Mo powder was influenced by multiple criteria. The use of pure elemental powders enables enhanced flexibility in customizing the composition and microstructure of the final alloy, a critical factor in maximizing unique properties. Moreover, pure powders enable the examination of the contributions of individual elements to the behavior of the alloy, offering a more comprehensive understanding of the material’s performance. Furthermore, this process facilitates the development of high-entropy alloys (HEAs) that offer distinctive properties, including improved mechanical strength and resistance to corrosion [20].

The powders were blended in a powder mixer for three hours to produce HEAs with the composition Cr_25-x_Co_25_Ni_25_Fe_25_Mo_x_ (x = 0, 5, 10%). Table 1 shows the weight percentages (wt%) of the powders. The substrate material used was SUS304, which measured 50 mm in width, 150 mm in length, and 25 mm in height.

**Figure 5:**
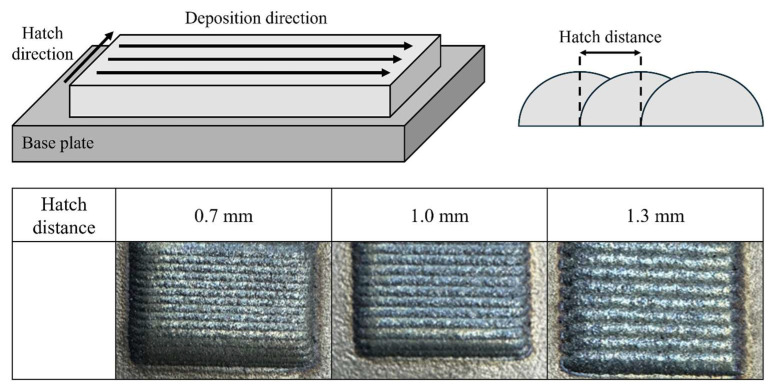
The initial experimental results about the hatching distance.

Table 2 shows the parameters of the DED procedure. The hatch distance is critical in determining the quality of the DED process [21,22]. If the hatch distance is too large, insufficient overlap can cause pores or cracks, reducing mechanical properties. Conversely, if the hatch distance is too small, the material’s height and edges may collapse [23]. Initial experiments were carried out to determine the ideal hatch distance. Figure 5 depicts the results of these experiments.

A 0.7 mm hatch distance produced good surface quality but an excessively high melt pool height of 1.25 mm, resulting in edge collapse. A 1.0 mm hatch distance resulted in a smooth surface and uniform stacking at a height of 1.05 mm. A 1.3 mm hatch distance had insufficient overlap, resulting in pores and a height of 0.78 mm. As a result, for this experiment, a 1.0 mm hatch distance was chosen to ensure good surface quality and uniform stacking. For tensile testing and microstructural analysis of Cr_25-x_Co_25_Ni_25_Fe_25_Mo_x_ (x = 0, 5, 10%), the workpiece was made in a rectangular shape with dimensions of 20 mm width, 120 mm length, and 8 mm height.

## 3. Results and Discussion

### 3.1. Microstructure

#### 3.1.1. X-ray Diffraction (XRD) Analysis

The crystal structure of Cr_25-x_Co_25_Ni_25_Fe_25_Mo_x_ (x = 0, 5, 10%) was determined via X-ray diffraction (XRD). The phase structure was identified using Cu Kα radiation (λ = 1.54 Å, 40 kV, 50 mA) on a Smart Lab SE from Rigaku Corporation, The Woodlands, TX, USA. 

**Figure 6:**
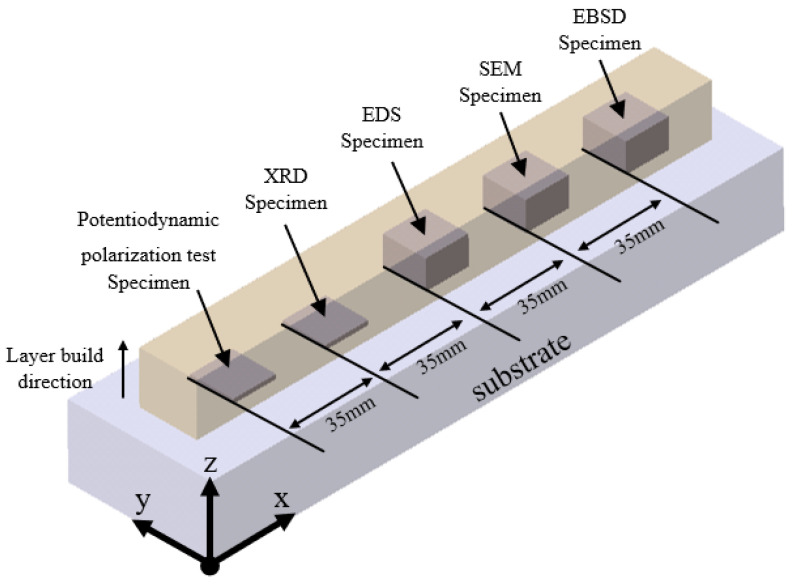
The extracted sampling positions for performing analysis.

**Figure 7:**
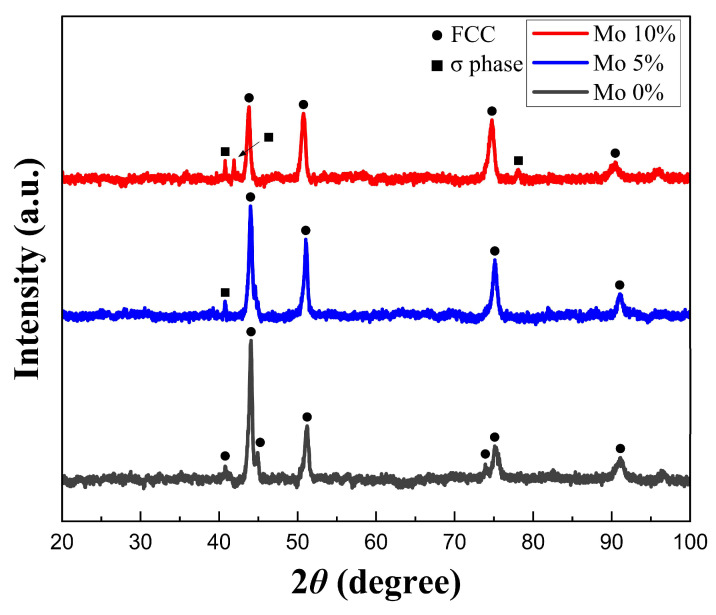
The XRD results of the Cr_25-x_Co_25_Ni_25_Fe_25_Mo_x_ (x = 0, 5, 10) specimen.

At 20 °C, XRD data were collected in 0.02° increments across a 2-theta range of 20–100°. Figure 6 depicts the locations where XRD, EBSD, SEM, EDS, and potentiodynamic polarization experiments were performed. The results of the XRD analysis are shown in Figure 7. The 0% Mo sample showed the FCC single-phase structure, while the 5% and 10% Mo samples showed (FCC + σ) dual-phase structure, which was caused by the interaction of Mo and Cr [12].

#### 3.1.2. Electron Backscatter Diffraction (EBSD) Analysis

**Figure 8:**
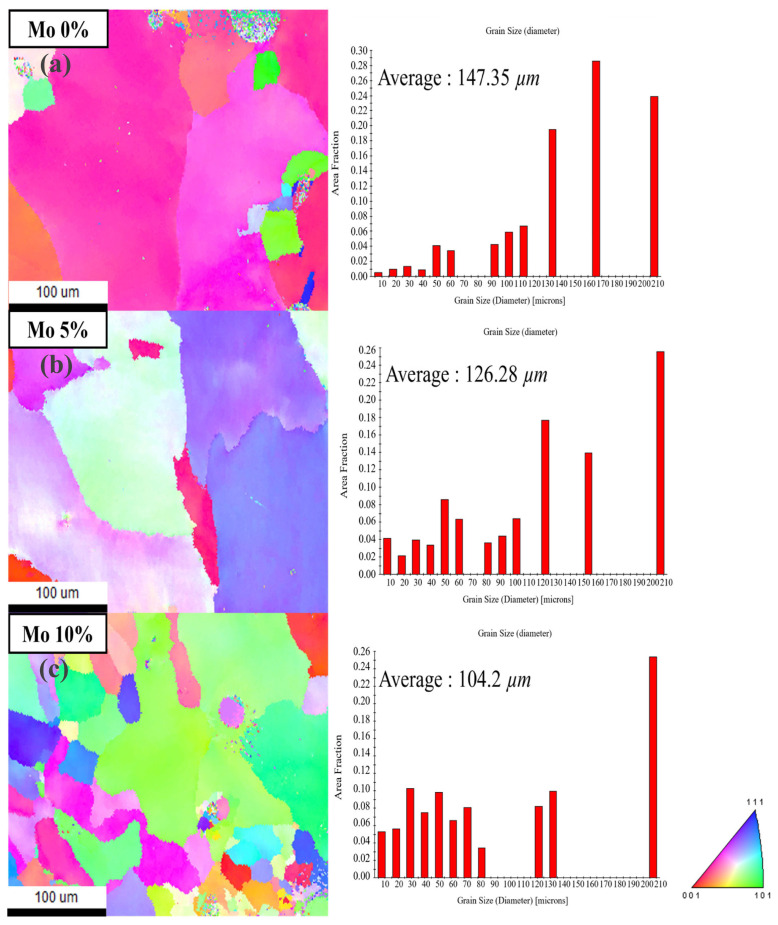
EBSD IPF mapping and grain size of (**a**) 0%, (**b**) 5%, and (**c**) 10% Mo specimen.

EBSD analysis was performed using an EBSD detector (Clarity, EDAX LLC, Mahwah, NJ, USA) to study the grain size and area fraction of the laminated Cr_25-x_Co_25_Ni_25_Fe_25_Mo_x_ (x = 0, 5, 10%). For the EBSD analysis, the middle section of the laminate was cut into 15 mm × 15 mm hatch pieces and 10 mm layer build pieces. These components were then mounted, polished, and silica coated. The EBSD investigation was carried out at the center of the cut specimens’ top surface. Figure 8 depicts the EBSD results for Cr_25-x_Co_25_Ni_25_Fe_25_Mo_x_ (x = 0, 5, 10%).

The inverse pole figure (IPF) shows that the 10% Mo sample had the smallest grain size. The average grain size for Cr_25-x_Co_25_Ni_25_Fe_25_Mo_x_ (x = 0, 5, 10%) was 147.35 µm, 126.28 µm, and 104.2 µm. The grains at 0% Mo were relatively large and made up of a single phase. However, at 5% and 10% Mo, the grain size decreased due to a grain refinement effect caused by the Mo addition. Furthermore, lattice mismatch occurs when the sizes and structural properties of Cr, Co, Ni, and Fe differ from those of the lattice medium. This lattice mismatch reduces grain growth and inhibits grain boundary movement, resulting in smaller grain sizes. As a result, adding Mo appears to increase grain boundary density and cause lattice mismatch, resulting in smaller grain sizes.

#### 3.1.3. Scanning Electron Microscopy (SEM) Analysis

**Figure 9:**
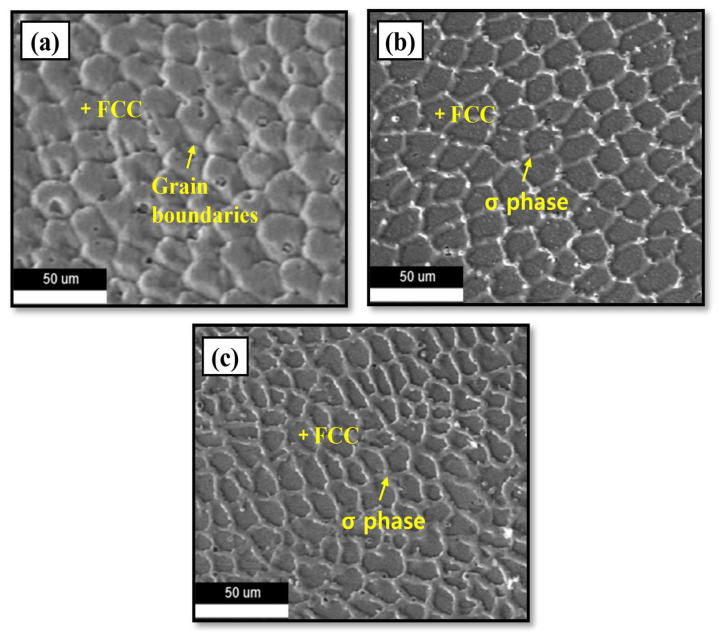
SEM image of (**a**) 0%, (**b**) 5%, and (**c**) 10% Mo specimen.

Using field emission scanning electron microscopy (FE-SEM) fitted with a Schottky emitter (MI-RA, TESCAN, Kohoutovice, Czech Republic), the microstructures of the Cr_25-x_Co_25_Ni_25_Fe_25_Mo_x_ (x = 0, 5, 10%) alloys were analyzed. Figure 9a demonstrates that the 0% Mo alloy showed an FCC phase characterized by a uniform solid solution structure. The microstructure exhibits well-defined grain boundaries, suggesting the existence of several grains within the FCC crystal phase. On the other hand, the 5% and 10% Mo alloys show that the addition of Mo resulted in the formation of the FCC phase as well as a secondary phase called the σ phase and presented a dual-phase structure (FCC + σ). 

**Figure 10:**
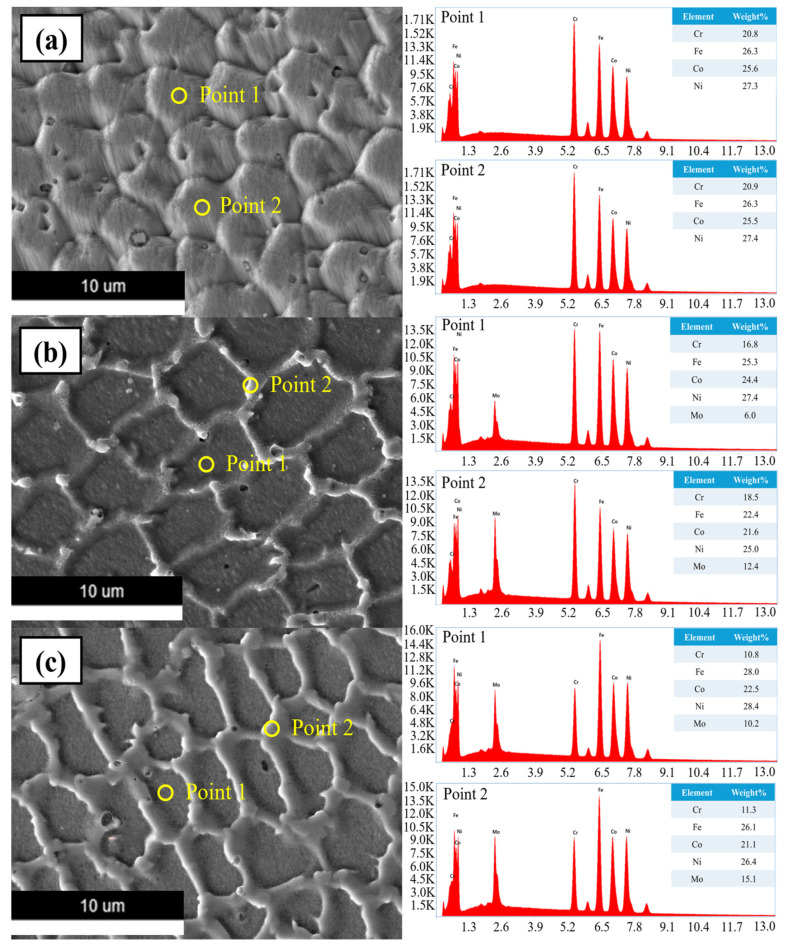
EDS results and a table analyzing the composition of each point of the (**a**) 0%, (**b**) 5%, and (**c**) 10% Mo specimen.

The addition of Mo enhances the chemical interactions and crystal nuclei of the alloy. The inclusion of Mo results in the creation of the σ phase, mainly as a consequence of the interactions between Cr and Mo. The phase development is enabled by the separation of Mo and Cr atoms at the grain boundaries, which serve as active sites for the initiation of the σ phase [16]. The confirmation of the σ phase is achieved by the conduct of XRD and EDS analyses, as depicted in Figure 7 and Figure 10, correspondingly.

The creation of the σ phase can take place by various mechanisms: The phenomenon of segregation at grain boundaries: Greater concentrations of Cr and Mo in the matrix can cause the development of the σ phase at the boundaries between grains. Nucleation sites for the σ phase are provided by this segregation. Direct precipitation occurs when the σ phase comes directly from the δ-ferrite phase (δ → σ) at the outer edge of δ islands.

The σ phase can also be generated by the eutectoid decomposition of δ-ferrite (δ → σ + γ), where γ represents a novel austenite phase. The nucleation and subsequent growth of the σ phase are regulated by thermodynamic driving forces and the diffusion of substitutional elements, namely Cr and Mo. Effective distribution of these elements is essential, since their ability to move and their concentration directly influence the locations where the σ phase precipitates [24].

The strong pinning influence of the σ phase on the movement of grain boundaries greatly improves the process of particle refinement. The pinning effect arises due to the obstruction of dislocation and grain boundary motion by the σ phase particles, resulting in the refinement of the alloy’s microstructure [25]. 

The presence of σ phase particles greatly improves the process of particle refinement by serving as physical obstacles that restrict the movement of dislocations and grain boundaries. This obstacle results in the buildup of dislocations at the σ phase particles, facilitating the creation of new grains and enhancement of the microstructure of the alloy. The σ phase particles specifically limit the movement of dislocations and grain boundaries by serving as obstacles, decreasing their ability to move [26,27].

Furthermore, the inclusion of Mo enhances this phenomenon. Mo atoms separate at the boundaries between grains and within the σ phase, enhancing the volume fraction and stability of the σ phase particles. This segregation intensifies the pinning effect, providing additional obstacles to the movement of dislocations and the migration of grain boundaries. Consequently, the growth in the quantity of σ phase particles caused by the inclusion of Mo results in a more significant restriction of dislocation and grain boundary motion [28,29].

Due to the combined effects of the σ phase and Mo addition, the alloy demonstrates enhanced mechanical strength and improved grain refinement. As seen in Figure 9, the SEM image depicts this expansion of the σ phase due to Mo addition.

#### 3.1.4. Energy-Dispersive X-ray Spectroscopy (EDS) Analysis

To determine the composition of the produced phases based on the Mo content, an EDS analysis was conducted using a silicon drift detector (Octane, EDAX) and SEM images for EDS. The EDS results are shown in Figure 10, accompanied by a table illustrating the composition at each location.

In Figure 10a, the EDS analysis shows that two points within the FCC phase exhibit similar compositions, suggesting a homogeneous distribution within the grain interior of the DED-fabricated high-entropy alloy. Nevertheless, the ratio of Cr to Mo is noticeably higher in Point 1 than in Point 2 when comparing the Point 1 FCC phase and Point 2 σ phase in Figure 10b,c. 

It can be concluded from this that the σ phase forms between the regions of the FCC solid solution after Cr and Mo interact chemically, and the FCC solid solution forms initially during the alloying process with Mo. Furthermore, a comparison of Figure 10b,c demonstrates that the σ phase width at Point 2 increases as the Mo content rises. As for precipitation, hardening causes grain boundary hardening and the σ phase to precipitate; therefore, a tensile strength test was performed.

### 3.2. Mechanical Properties

#### 3.2.1. Tensile Strength

**Figure 11:**
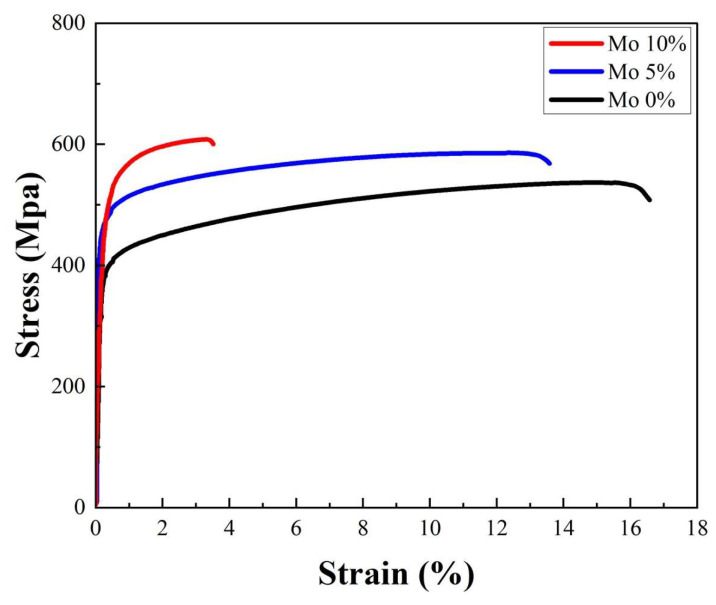
Tensile test results of Cr_25-x_Co_25_Ni_25_Fe_25_Mo_x_ (at x = 0, 5, 10%) specimen.

A tensile strength test was performed to evaluate the effect of σ phase precipitation on the tensile strength of Cr_25-x_Co_25_Ni_25_Fe_25_Mo_x_ alloys (x = 0, 5, 10%). The yield strengths (YSs) and ultimate tensile strengths (UTSs) were determined using a tensile testing machine (Z250, ZwickRoell, Ulm, Germany) with a maximum test force of 20 tons. The specimens were made in a dog bone shape under ASTM E8/E8M [30] specifications. Figure 11 depicts the tensile strength test results for the Cr_25-x_Co_25_Ni_25_Fe_25_Mo_x_ alloys (x = 0, 5, and 10%).

The YS and UTS of the 0% Mo specimen were measured to be 388 MPa and 524 MPa, respectively. On the other hand, the 5% Mo specimen exhibited YS and UTS values of 466 MPa and 574 MPa, respectively. These values indicate 20.1% and 9.5% improvements compared to the 0% Mo specimen. The 10% Mo specimen offered YS and UTS values of 514 MPa and 631 MPa, respectively. These values indicate increases of 32.5% and 20.4% compared to the 0% Mo specimen and improvements of 10.3% and 9.9% among the 5% Mo specimen. These findings demonstrate that increasing the amount of Mo in this alloy enhances the YS and UTS indices. This is because increased σ phase precipitation at the grain boundaries enhances the resistance to dislocation movement, which makes the alloy stronger overall [16]. Furthermore, the elongation of the 5% Mo and 10% Mo specimens was 17.7% and 76.6% less than those of the 0% Mo specimens, respectively. As the Mo content increased, the elongation decreased. 

**Figure 12:**
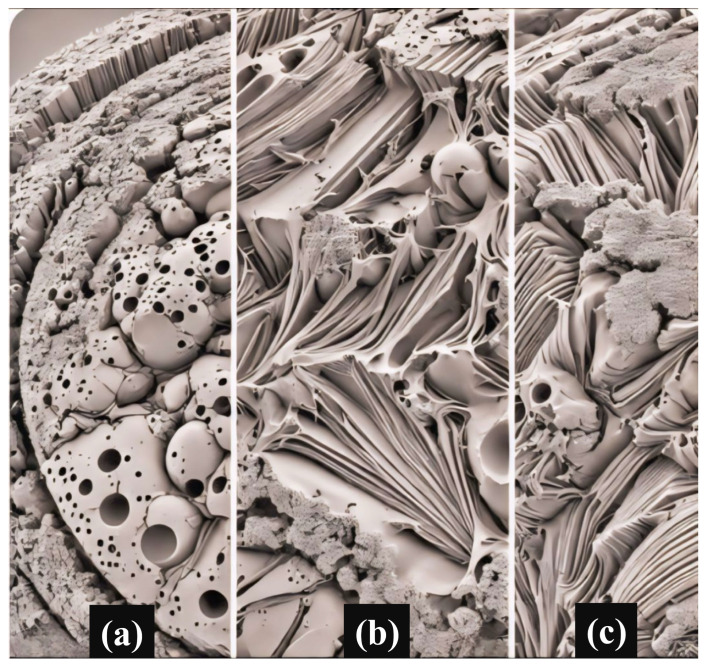
SEM image of fracture surfaces of (**a**) 0%, (**b**) 5%, and (**c**) 10% Mo specimen.

As seen in Figure 12, the SEM image of fracture surfaces from three alloys with different Mo concentrations (0%, 5%, and 10%) offers essential information about the observed elongation behavior during tensile testing. The SEM study shows a notable evolution in fracture properties related to the rise in Mo content. The 0% Mo alloy exhibits a ductile fracture morphology, defined by a relatively smooth surface with dimples. This morphology suggests that the alloy has excellent plasticity and high extendability. As shown by the presence of dimples alongside some sharp fractures, the 5% Mo alloy displays a mixed fracture mode that combines both ductile and brittle characteristics. This shift implies that the incorporation of a moderate quantity of Mo starts to impact the structural integrity of the alloy. 

The 10% Mo alloy primarily exhibits a brittle fracture appearance, distinguished by a rougher surface with less well-defined depressions and more prominent crack propagation routes. The observed decrease in elongation of 17.7% and 76.6% less than the 0% Mo specimens for the 5% and 10% Mo alloys, respectively, correlates with the shift in fracture mechanism. This indicates that as the Mo content reaches higher levels, the alloy becomes more brittle and less ductile. These findings indicate that an excessive amount of Mo added to the alloy changes its microstructural properties and ultimately affects its mechanical performance. This highlights the need to maintain a proper balance in alloy composition to attain the desired mechanical characteristics.

In conclusion, the results of the tensile test show that the Cr_25-x_Co_25_Ni_25_Fe_25_Mo_x_ (x = 0, 5, 10%) alloys exhibit significant increases in the YS and UTS when the Mo content is raised. Nevertheless, this results in less elongation, indicating a trade-off between ductility and strength. The main cause of these modifications is the higher Mo content, which increases the σ phase precipitation and strengthens the alloy overall by improving its resistance to dislocation movement.

#### 3.2.2. Potentiodynamic Polarization Test

**Figure 13:**
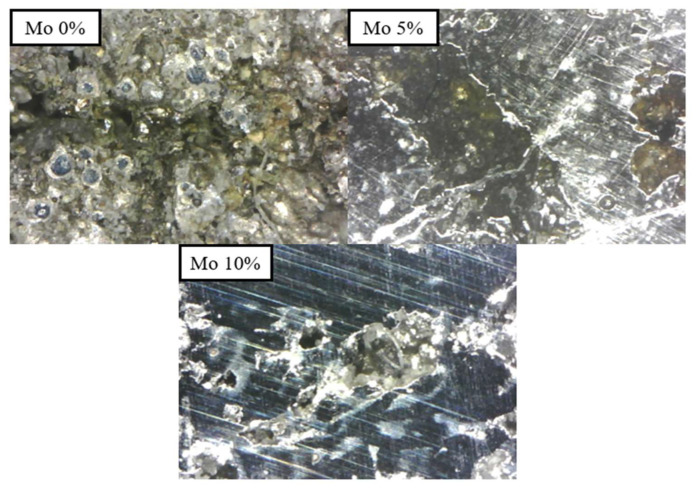
The micrograph of the specimen results of corrosion tests.

The corrosion behavior of Cr_25-x_Co_25_Ni_25_Fe_25_Mo_x_ (x = 0, 5, 10%) alloys was assessed through potentiodynamic polarization testing. Potentiostat (SP-200, Biologic, Seyssinet-Pariset, France) tests were performed at room temperature (20 °C) in a 3.5 wt% NaCl solution. To ensure accuracy, the potentiodynamic polarization curves were measured at least three times. The micrographs of the specimens after the corrosion test are shown in Figure 13, which indicates the resistance to pitting corrosion in the specimens improved gradually as the Mo content increased (0%, 5%, and 10% Mo).

**Figure 14:**
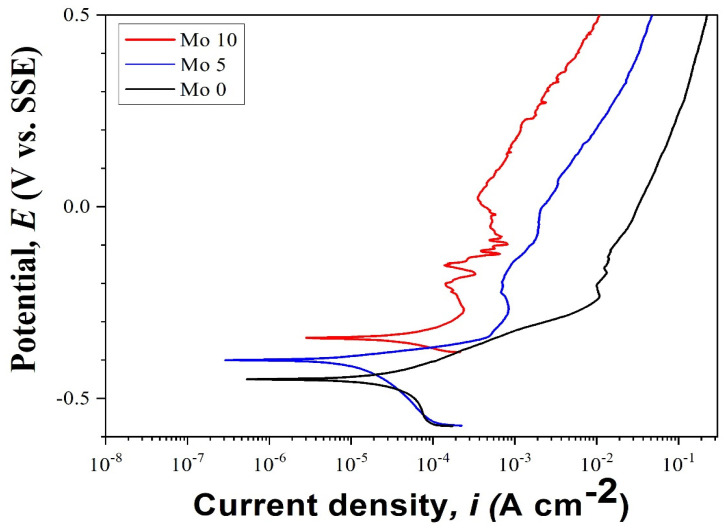
Potentiodynamic polarization test curves of Cr_25-x_Co_25_Ni_25_Fe_25_Mo_x_ (at x = 0, 5, 10%) specimen.

Figure 14 presents the potentiodynamic polarization curves for each condition. The corrosion potential (E_corr_) indicates resistance to early corrosion, with higher E_corr_ values signifying greater resistance. The corrosion current density (I_corr_) represents the rate of corrosion progression, with higher I_corr_ values indicating faster corrosion. Due to its high current density and low corrosion potential, the 0% Mo alloy was subject to rapid initial corrosion. 

The 5% Mo alloy displayed a higher potential and lower current density than the 0% Mo alloy, suggesting greater resistance to initial corrosion and a slower progression of corrosion. The highest corrosion potential and the lowest current density were observed in the 10% Mo alloy, which subsequently exhibited the highest resistance to initial corrosion and the slowest corrosion progression. Consequently, according to the parameters of that study, an increase in Mo content is associated with a slower progression of corrosion and higher resistance to initial corrosion.

## 4. Conclusions

This study included the fabrication of Cr_25-x_Co_25_Ni_25_Fe_25_Mo_x_ (at x = 0, 5, 10%) HEAs utilizing the DED process. The deposition process included the mixture of the individual powders, followed by their application onto a SUS304 substrate. A comprehensive investigation of the manufactured alloys’ microstructure, mechanical characteristics, and corrosion behavior was carried out. The results are explained in brief as follows:

1. An initial experiment was conducted to obtain the ideal hatch distance for Cr_25-x_Co_25_Ni_25_Fe_25_Mo_x_ (at x = 0, 5, 10%). The findings demonstrated that a hatch distance of 1.0 mm was optimal since it achieved homogeneity without excessive overlap or edge collapse. Reduced uniformity was observed while using a hatch distance of 1.3 mm, but a hatch distance of 0.7 mm resulted in increased height due to excessive overlap, which ultimately caused edge collapse.
2. XRD investigation verified the existence of the FCC single-phase structure in the 0% Mo specimen, whereas the 5% and 10% Mo samples exhibited the creation of the FCC + σ dual-phase structure as a result of the interaction between Mo and Cr. Analysis using EBSD revealed that the average grain sizes for 0%, 5%, and 10% Mo were 147.35 µm, 126.28 µm, and 104.2 µm, respectively. This indicates that the grain size decreases as the Mo concentration increases. The creation of the FCC + σ dual-phase structure was identified as the cause of this grain refinement. SEM investigation demonstrated that the presence of Mo resulted in the formation of the σ phase between FCC phases, as observed using electron microscopy. The volume fraction of the σ phase increased proportionally with the Mo level. The chemical composition differences between the FCC and σ phases were confirmed by EDS analysis, which indicated the interaction between Mo and Cr in the production of the σ phase.
3. Using Mo greatly enhanced the mechanical properties of the alloys. The inclusion of Mo facilitated the formation of the σ phase, resulting in an enhanced grain boundary-strengthening effect and improved mechanical strength. The results of the tensile test demonstrated that the inclusion of Mo increased YS and UTS. The YS and UTS of the specimens with 0% Mo were measured to be 388 MPa and 524 MPa, respectively. By including 5% Mo, the YS and UTS experienced significant enhancements, reaching 466 MPa and 574 MPa, respectively. These increases correspond to a 20.1% increase in YS and a 9.5% increase in UTS. By including 10% Mo, the YS and UTS were elevated to 514 MPa and 631 MPa, respectively. This corresponds to 32.5% and 20.4% enhancements compared to the alloy with 0% Mo.
4. The corrosion resistance of HEAs was determined by applying potentiodynamic polarization tests in a 3.5-weight percent sodium chloride (NaCl) solution. The results showed that the inclusion of Mo significantly improved the initial corrosion resistance of the alloys. The 5% Mo alloy demonstrated a higher E_corr_ and a lower I_corr_ than the 0% Mo alloy, suggesting a superior ability to resist initial corrosion. The 10% Mo alloy exhibited the highest corrosion potential and the lowest current density, leading to the slowest corrosion growth and the highest resistance to initial corrosion.

Thus, this study observed that higher Mo content is associated with better mechanical properties and increased resistance to initial corrosion.

## Figures and Tables

**Table 1:** Chemical compositions of DED process Cr_25-x_Co_25_Ni_25_Fe_25_Mo_x_ (x = 0, 5, 10) alloys (wt%).

Alloy	Cr	Co	Ni	Fe	Mo
Mo0	23.06	26.14	26.03	24.77	-
Mo5	17.76	25.16	25.06	23.84	8.19
Mo10	12.84	24.25	24.15	22.98	15.79

**Table 2:** The DED conditions.

Process Parameter	Value
Laser power (W)	1000
Scanning speed (mm/s)	10
Shielding gas flow (L/min)	25
Powder feed rate (g/min)	7
Hatch distance (mm)	1.0

## Data Availability

The authors confirm that the data supporting the findings of this study is available within the article.
